# Lutein Dietary Supplementation Attenuates Streptozotocin-induced testicular damage and oxidative stress in diabetic rats

**DOI:** 10.1186/s12906-015-0693-5

**Published:** 2015-06-30

**Authors:** Amal J. Fatani, Salim S. Al-Rejaie, Hatem M. Abuohashish, Abdullah Al-Assaf, Mihir Y. Parmar, Mohammed M. Ahmed

**Affiliations:** Department of Pharmacology and Toxicology, College of Pharmacy, King Saud University, P.O. Box 55760, Riyadh, 11544 Saudi Arabia; Department of Science and Nutrition, College of Food and Agricultural Sciences, King Saud University-11544, Riyadh, Saudi Arabia; Department of Biomedical Dental Sciences, College of Dentistry, University of Dammam, Dammam-31441, Saudi Arabia

**Keywords:** Lutein, Diabetes mellitus, Oxidative stress, Testicular cells

## Abstract

**Background:**

Diabetes mellitus with the successive generation of reactive oxygen species signifies a major risk factor for testicular dysfunction. Antioxidant supplements are one of the best options to prevent such disorder. In the present study, lutein as dietary supplement has been used to explore its potential protective effects against diabetes-induced oxidative stress in testicular cells.

**Methods:**

Diabetes was induced using a single IP injection of streptozotocin (STZ). Lutein was mixed with rat chow powder and supplemented to diabetic rats for 5 weeks. Serum testosterone levels were estimated. In testicular cells, thiobarbituric acid reactive substances (TBARS), total sulfhydryl groups (T-GSH), non-protein sulfhydryl groups (NP-SH), superoxide dismutase (SOD) and catalase (CAT) activities were measured. Pro-inflammatory mediators like tumor necrosis factor α (TNF-α) and interleukin 1β (IL-1β) were measured in the testis. Nucleic acids and total protein (TP) levels were also estimated in testicular cells. Histopathological changes were evaluated in testis.

**Results:**

Serum testosterone level was significantly decreased in diabetic animals compared to controls. Diabetes markedly reduced T-GSH, NP-SH, CAT and SOD, while TBARS, TNF-α and IL-1β levels were increased in the diabetic testis compared to non-diabetic controls. Lutein supplementation, significantly and dose dependently increased the serum testosterone level. The elevated TBARS levels were significantly decreased compared to diabetic group, while the decreased levels of T-GSH and NP-SH and activities of CAT and SOD were found increased by lutein treatments in dose dependent manner. Lutein pretreatment also inhibited the TNF-α and IL-1β levels compared to diabetic group. The decreased values of nucleic acids and total protein in diabetic group were also significantly increased in lutein supplemented groups. The histopathological evaluation revealed protection the damaged testicular cells in the diabetic rats by lutein supplementation.

**Conclusion:**

These findings showed that lutein has potential beneficial effects in diabetes-induced testicular damage, probably through its antioxidant and anti-inflammatory properties.

## Background

Male reproductive alterations have been widely reported in experimental animal model and human with diabetes [1]. Streptozotocin (STZ) induced diabetes in male rats results in atrophy of sex organ, changes in histoarchitecture of ventral prostate [2], decrease in sperm count [3], along with low levels in plasma testosterone [4]. Oxidative stress is one of the major pathophysiological routes during diabetes mellitus (DM) [5, 6]. Persistent hyperglycemia leads to the formation of advanced glycation end-products (AGEs) which are the products of non-enzymatic reactions between glucose and lipids, proteins or nucleic acids [7]. AGEs and glucose auto-oxidation might contribute to diabetes-induced sexual dysfunction by generating oxygen free radicals especially reactive oxygen species (ROS), which induce oxidative cellular damage and quench nitric oxide (NO), terminating in decreased cyclic guanosine monophosphate (cGMP) and impairing cavernosal smooth muscle relaxation [7]. Furthermore, ROS are positively correlated with both insulin resistance and the deterioration of cell function in the context of concomitant hyperglycemia [6, 8] and their cytotoxic effects are usually accompanied with an increase in lipid peroxidation, alteration of the glutathione redox state, a decrease in the content of individual natural antioxidants, and decreased induction of antioxidant enzymes [9]. Several studies reported hyperglycemia-induced elevation of proinflammatory cytokines in serum of diabetic individuals [10, 11]. This elevation was abolished by supplementation of the antioxidants suggesting that hyperglycemia-induced cytokine production may be mediated by ROS [12].

Fruits and vegetables contain a vast array of antioxidant components, which possess several physiological properties including, protection against oxidative stress-mediated diseases. Lutein is one of those most widely distributed carotenoids in fruits and vegetables having antioxidant properties [13]. It is not naturally produced by the human body; therefore, it must be obtained through the consumption of foods containing large amount of lutein such as dark-green leafy vegetables kale, spinach, turnip greens, and collards [14]. A number of recent studies have discussed the protective effects of lutein against different eye diseases, mainly age-related macular degeneration, correlating the reported to the antioxidant property of lutein [15–17]. The mechanisms of antioxidant activity of lutein is well established and extensively described in the literature [16, 18].

Diabetes-induced sexual dysfunction is considered one of the most prevalent diabetic complications where oxidative stress and inflammation are deemed to play vital role in its pathogenesis. It can be anticipated that lutein via its reported antioxidant and anti-inflammatory properties may protect the diabetes-induced testicular damage from the deleterious effects of ROS and inflammation. Thus, the study was designed to investigate the protective ability of lutein on diabetic associated testicular dysfunction in STZ animal model of DM.

## Methods

### Animals

Male Wistar albino rats (12 to 13 weeks old) weighting 260 ± 10 g were received from Experimental Animal Care Center, College of Pharmacy, King Saud University, Riyadh, Saudi Arabia. All animals were maintained under controlled conditions of temperature (22 ± 1 °C), humidity (50-55 %), light (12 h light/12 h dark cycle) with free access to Purina rat chow (Manufactured by Grain Silos & Flour Mills Organization, Riyadh, Saudi Arabia) and drinking water. Animals were acclimatized to the laboratory conditions for 7 days. All experimental procedures including handling, treatment and euthanasia were conducted in accordance with the National Institute of Health Guide for the Care and Use of Laboratory Animals (NIH Publications No. 80–23; 1996) as well as the Ethical committee of Experimental Animal Care Center, College of Pharmacy, King Saud University, Riyadh, Saudi Arabia obtained approval (238-EACC-2014).

### Diet

Experimental diets were prepared in pellet form by adding lutein in three different doses 40 mg/kg, 80 mg/kg and 160 mg/kg in rat chow powder following shade dry method. The above doses are calculated on the bases of food intake of diabetic rats as 35 to 40 g per day, thus the range of our doses will be in between 1.5 to 6 mg/day. Lutein was purchased from Carbone Scientific Co., Ltd (Product number C-23248, London, UK). During whole experimental period, all groups of animals were kept on free access to food and water.

### Diabetes induction

Induction of experimental diabetes was done using single intraperitoneal injection of STZ (Sigma) dissolved in citrate buffer (pH 4.5) at the dose of 65 mg/kg to overnight fasted rats. Control animals received intraperitoneal injection of citrate buffer only as vehicle. Forty-eight hours later, animals were fasted and blood glucose levels were analyzed using a glucometer (ACCU-CHEK ACTIVE, Roche, Germany). Animals with blood glucose levels above 250 mg/dL were considered as diabetic.

### Experimental design

Six normal healthy rats were taken in control group and diabetic rats were randomly divided into four groups by taking six in each, as follows;

1. Non-Diabetic (ND)
2. Diabetic (D)
3. Diabetic supplemented with lutein (40 mg/kg diet) (D + L40)
4. Diabetic supplemented with lutein (80 mg/kg diet) (D + L80)
5. Diabetic supplemented with lutein (160 mg/kg diet) (D + L160)

Lutein content diets were supplemented at free access to diabetic rats for 5 weeks. The general health of the animals was observed during treatment period and their body weights were recorded at the beginning of every week throughout the study period. At the end of the 5^th^ week, blood samples were collected through the cardiac puncture under light anesthesia. Animals were then sacrificed and their testicular tissues were dissected and stored at −80 °C till analysis. A small testicular cross section from each group was fixed in 10 % formaldehyde solution for histopathological evaluation.

### Estimations of glucose, insulin and testosterone levels in serum

Serum levels of glucose were measured using the commercially available kit (RANDOX Laboratories Ltd., UK) while insulin and testosterone levels in serum were assayed using ELISA kit (Cayman Ltd., USA) according to the manufacturer’s instructions.

### Estimations of TNF-α and IL-1β levels in testicular cells

Proinflammatory cytokines including TNF-α and IL-1β levels in testicular cells were assessed and quantified (pg/mg protein) by using ELISA technique (R & D systems, USA).

### Estimation of TBARS levels in testicular cells

Levels of thiobarbituric acid reactive substances (TBARS) were estimated using biochemical assay kit (ZeptoMetrix) by measuring the lipid peroxidation products, malondialdehyde (MDA) equivalents. 0.1 mL of testis homogenate was mixed with 2.5 mL reaction buffer (provided by the kit) and heated at 95 °C for 60 min. After cooling, the absorbance of the supernatant was measured at 532 nm. The quantified MDA levels were expressed as nmole/mg protein.

### Estimations of T-GSH and NP-SH levels in testicular cells

Total sulfhydryl group levels were estimated following the method described by Sedlak and Lindsay [19]. Testis homogenate was mixed with 0.2 M Tris buffer (pH 8.2), 0.01 M Ellman’s reagent and 5,5’-dithiobis-(2-nitro-benzoic acid) (DTNB). After centrifugation at room temperature, the absorbance of the clear supernatants was measured at 412 nm. For non-sulfhydryl group estimations, testis homogenate was mixed with 50 % trichloroacetic acid solution (TCA). Then after shaking intermittently for l0-15 min, samples were centrifuged for 15 min and 2 mL of the supernatant was mixed with 4 mL of 0.4 M Tris buffer (pH 8.9) and 0.1 ml DTNB. The absorbance was read within 5 min at 412 nm.

### Assessment of enzymatic activities SOD and CAT in testicular cells

Testicular SOD activity was estimated following the method described by Kono, [20]. Superoxide anions were generated by the oxidation of hydroxylamine hydrochloride. The reduction of nitroblue tetrazolium to blue formazan mediated by superoxide anions was measured at 560 nm under aerobic conditions. Addition of SOD inhibited the reduction of nitroblue tetrazolium and the extent of inhibition was taken as a measure of enzyme activity. Testicular CAT activity was estimated following the method described by Aebi, [21]. Briefly, 0.5 mL of the post-mitochondrial supernatant was mixed with 50 mM phosphate buffer (pH 7.0) and 20 mM H2O2. The estimation was done using spectrophotometer following the decrease in absorbance at 240 nm.

### Estimation of nucleic acids and total protein (TP) levels in testicular cells

Nucleic acids in testicular cells were estimated following the method described by Bregman, [22]. In brief, homogenates were suspended in 10 % ice-cold trichloroacetic acid (TCA). Then after centrifugation, pellets were extracted with 95 % ethanol twice. Nucleic acids extract was treated either with diphenylamine or orcinol reagent for DNA and RNA quantification respectively. The modified Lowry method by Schacterle and Pollack, [23] was used to estimate levels of TP in testis using bovine plasma albumin as a standard.

### Histopathological assessment of the testicular tissues

Testicular cross sections fixed in 10 % formaldehyde solution then embedded into paraffin wax blocks and cut using a microtome. Samples were stained with haematoxylin and eosin stain (H&E), mounted and observed microscopically by a histopathologist in blinded fashion to avoid any kind of bias.

### Statistical analysis

Data were expressed as mean ± standard error of mean (SEM) and statistically analyzed using one-way ANOVA followed by Student-Newman-Keuls multiple comparisons test. Graph Pad prism program (version 5) was used as analyzing software.

## Results

### Effects on fasting blood glucose, insulin levels and animals body weights

Fasting glucose and insulin levels were significantly increased and decreased in diabetic animals compared to control rats respectively. These changes were not markedly altered with the lutein dietary (40, 80 and 160 mg/kg diet) supplementations. Mean body weights and testis weight compared to body weights (g/100 g body weight) of diabetic rats found significantly decreased. The experimental diets could not alter the body and testis weights in diabetic rats compared to normal diet fed diabetic rats (Table 1).

**Table 1:** Effect of lutein on body and organ weight in of diabetic animals

Treatments	Glucose (dl/L)	Insulin (ng/ml)	Body weight (g)		Testis weight g/100 g
			Initial	Final	
ND	130.83 ± 3.28	25.65 ± 3.51	215.33 ± 5.01	327.17 ± 13.08	0.10 ± 0.09
D	526.17 ± 27.81^*a^	11.65 ± 1.21^*a^	202.00 ± 4.96	190.33 ± 14.72^*a^	0.50 ± 0.12^*a^
D + L(40)	507.17 ± 16.11	12.56 ± 2.54	224.87 ± 6.57	198.00 ± 6.92	0.67 ± 0.15
D + L(80)	493.83 ± 23.36	13.24 ± 2.16	232.28 ± 3.69	189.5 ± 9.13	0.75 ± 0.16
D + L(160)	465.83 ± 28.17	13.68 ± 2.07	223.28 ± 6.10	179.67 ± 7.51	0.85 ± 0.07

Data were expressed as Mean ± SEM. and analyzed using one-way ANOVA followed by Student-Newman-Keuls multiple comparisons test (*n* = 6). Difference between groups was considered statistically significant when ^(*)^ P ≤ 0.05. ^(a)^ D group was compared with ND group

### Effects on serum testosterone levels

**Fig. 1:**
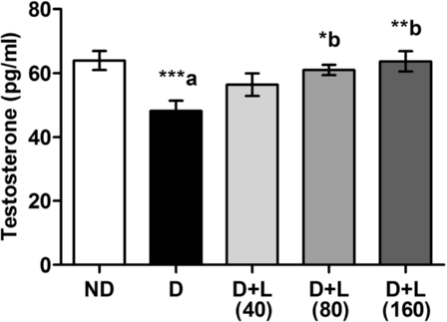
Effect of lutein on serum level of testosterone in diabetic rats. Note the lutein dose dependent for monitoring the level of testosterone up to ND group. Data were expressed as Mean ± SEM. and analyzed using one-way ANOVA followed by Student-Newman-Keuls multiple comparisons test (*n* = 6). Difference between groups was considered statistically significant when ^(*)^P ≤ 0.05, ^(**)^P ≤ 0.01 and ^(***)^P ≤ 0.001. ^(a)^D group was compared with ND group; ^(b)^lutein treated groups were compared with D group

Serum levels of testosterone showed a significant decrease in diabetic animals compared to control rats. Five weeks of lutein (80 and 160 mg/kg) diets supplementation revealed significant increase in decreased levels of serum testosterone (Fig. 1).

### Effects on testicular pro-inflammatory cytokines

**Fig. 2:**
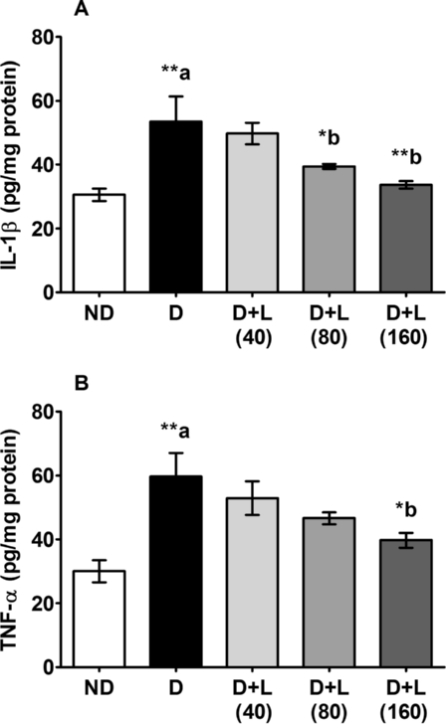
Effect of lutein on testicular levels of (**a**) IL-1β and (**b**) TNF-α of diabetic rats. Note the down regulation of both cytokines on the lutein dose- dependent groups was compared to D group. Data were expressed as Mean ± SEM. and analyzed using one-way ANOVA followed by Student-Newman-Keuls multiple comparisons test (*n* = 6). Difference between groups was considered statistically significant when ^(*)^ P ≤ 0.05 and ^(**)^ P ≤ 0.01. ^(a)^D group was compared with ND group; ^(b)^lutein treated groups were compared with D group

Testicular levels of pro-inflammatory cytokines including TNF-α and IL-1β were significantly increased in diabetic rats compared to control animals. Lutein supplementation with medium and higher doses (80 and 160 mg/kg) to diabetic rats for five weeks revealed inhibition in IL-1β while TNF-α levels decreased only in 160 mg/kg lutein diet supplemented group when compared to normal diet fed diabetic rats (Fig. 2).

### Effects on testicular lipid peroxidation and oxidative stress

**Fig. 3:**
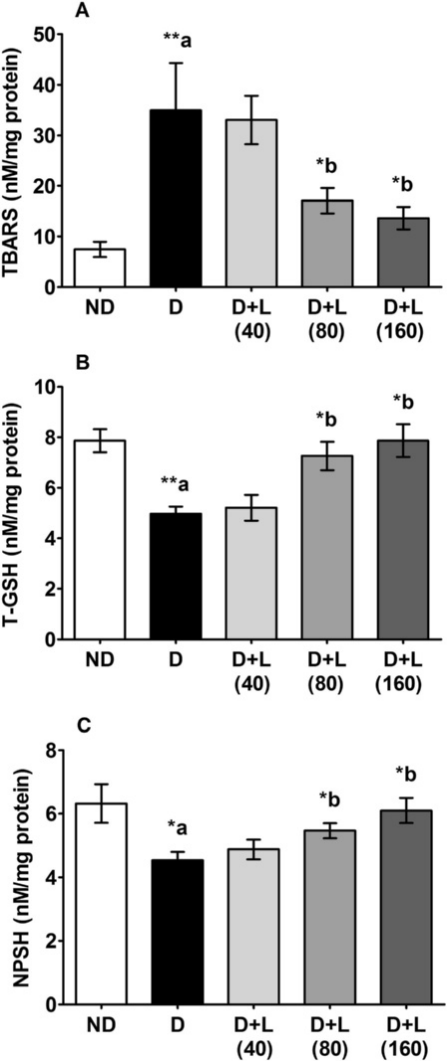
Effect of lutein on testicular levels of (**a**) TBARS, (**b**) T-GSH and (**c**) NPSH of diabetic rats. Note a significant decreased of TBARS in group treated lutein (160 mg) comparing to diabetic one, while highly significant increased was noticed in the T-GSH and NPSH. Data were expressed as Mean ± SEM. and analyzed using one-way ANOVA followed by Student-Newman-Keuls multiple comparisons test (*n* = 6). Difference between groups was considered statistically significant when ^(*)^ P ≤ 0.05 and ^(**)^ P ≤ 0.01. ^(a)^ D group was compared with ND group; ^(b)^ lutein treated groups were compared with D group

In testicular tissues of diabetic animals, TBARS levels found significantly increase as compared to control group of rats. Lutein dietary supplementation to diabetic rats with medium and higher doses revealed significant inhibition in the elevated levels of TBARS as compared to normal diet fed diabetic rats. In sulfhydryl groups, both T-GSH and NP-SH levels markedly reduced in testicular cells of diabetic animals compared to healthy animals. Supplementations with the medium and high doses of lutein content diets (80 and 160 mg/kg) to diabetic rats significantly ameliorated the reduced levels of testicular T-GSH and NPSH in diabetic rats (Fig. 3).

**Fig. 4:**
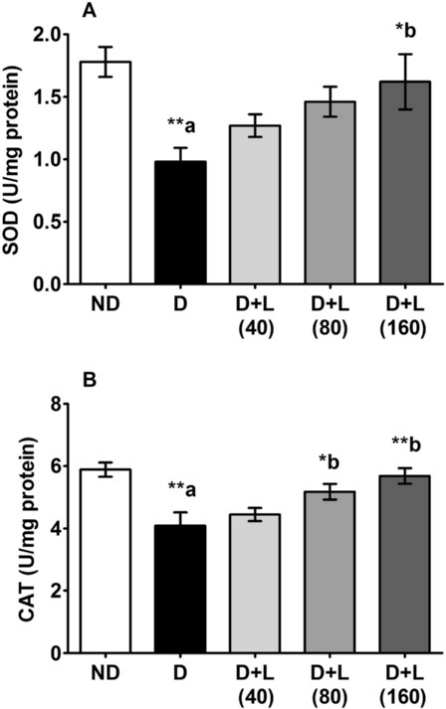
Effect of lutein on testicular activities of (**a**) SOD and (**b**) CAT of diabetic rats. Note the up regulating of both enzymes on the lutein dose dependent groups comparing to D group. Data were expressed as Mean ± SEM. and analyzed using one-way ANOVA followed by Student-Newman-Keuls multiple comparisons test (*n* = 6). Difference between groups was considered statistically significant when ^(*)^ P ≤ 0.05 and ^(**)^ P ≤ 0.01. ^(a)^D group was compared with ND group; ^(b)^lutein treated groups were compared with D group

Testicular antioxidant enzymes (SOD and CAT) activities were decreased significantly in diabetic animals as compared to control group of rats. Five consecutive weeks of treatment with the medium and higher doses of Lutein (80 and 160 mg/kg/day) significantly enhanced the reduced activities of CAT as compared to diabetic group (Fig. 4), while only the higher dose of lutein (160 mg/kg/day) significantly inhibited the STZ-induced decrease in SOD testicular activity (Fig. 4).

### Effects on testicular nucleic acids and total proteins

**Fig. 5:**
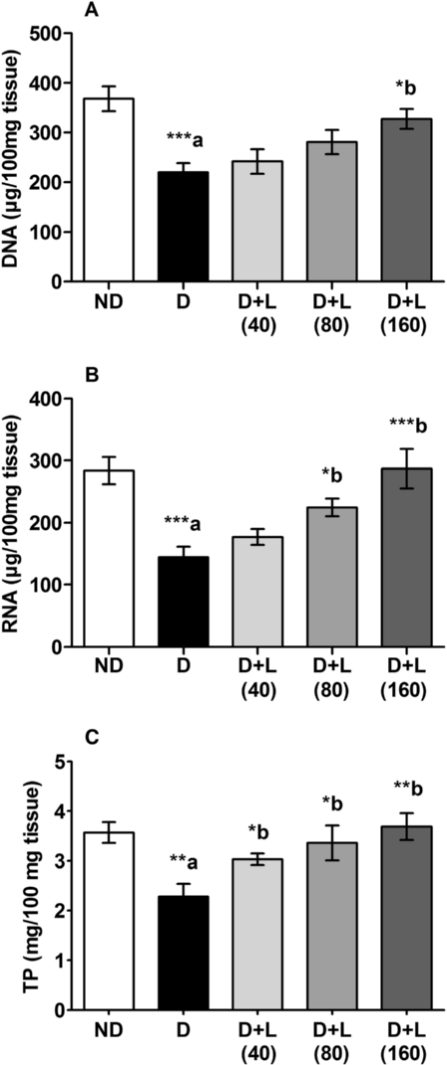
Effect of lutein on testicular levels of (**a**) DNA, (**b**) RNA and (**c**) TP of diabetic rats. Note the lutein dose- dependent of monitoring the expression of DNA, RNA and TP were increased up to ND group. Data were expressed as Mean ± SEM. and analyzed using one-way ANOVA followed by Student-Newman-Keuls multiple comparisons test (*n* = 6). Difference between groups was considered statistically significant when ^(*)^ P ≤ 0.05, ^(**)^ P ≤ 0.01 and ^(***)^P ≤ 0.001. ^(a)^D group was compared with ND group; ^(b)^lutein treated groups were compared with D group

There was a significant decrease in testicular DNA, RNA and TP concentrations in the diabetic groups of animals as compared to normal animals (Fig. 5). The reduced levels of testicular DNA were significantly attenuated by both the medium and higher doses of lutein (80 and 160 mg/kg), while only the higher dose of lutein (160 mg/kg/day) brought back RNA levels to its normal values (Fig. 5). The reduced levels of TP was significantly increased in lutein supplemented groups as compared to untreated diabetic rats (Fig. 5).

### Effects on testicular histopathological features

**Fig. 6:**
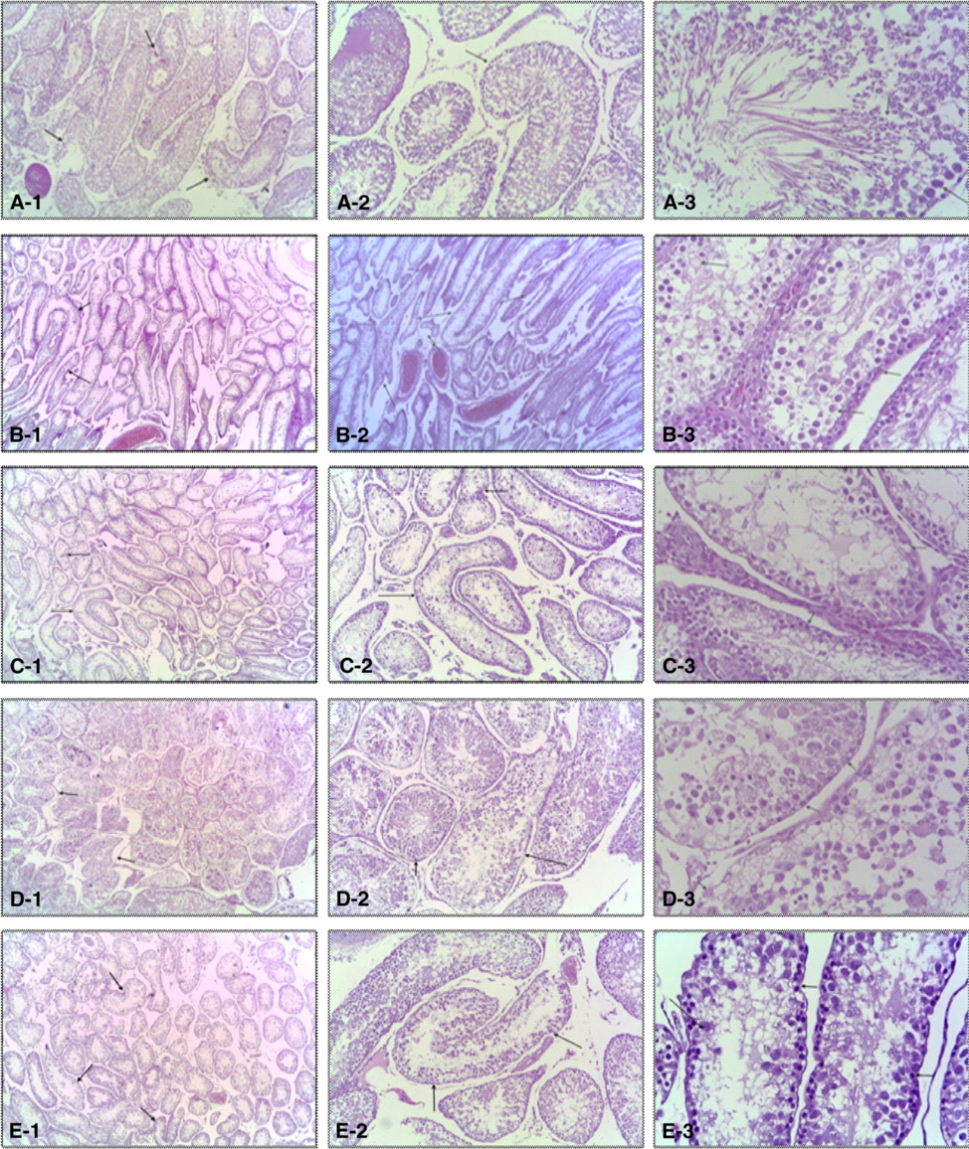
Histopathological sections of testis from rats stained with H&E (4, 10 and 40X; respectively). Testicular microscopic image of [**a**] Normal rat testis from ND group; [**b**] Diabetic rat testis with degenerated and edematous spermatogonia cells with dilated and congested blood vessels; [**c**, **d & e**] dose dependent reparative testicular changes and edematous healing lutein treated rats (40, 80 and 160 mg/kg, respectively)

Histopathological investigation of the ND group revealed germinal epithelium forms about 90 % and Stromal about 10-15 %. The germinal cells are complete up to spermatids and full sperm maturation. The interstitial tissue shows a full spermatogenesis (Fig. 6 a-1, A-2 and A-3). In the D group, there was a marked thickening of the basement membrane and abnormal configurations with elongation of the tubules with variable in size and contour. The spermatogonia cells show degeneration in most of the tubules while they are proliferating in others. Also, the maturation is up to primary spermatogonia and the inter stitial tissue was edematous and contains numerous thickened wall dilated and congested blood vessels (Fig. 6 b-1, b-2 and b-3). Results of the D + L40 group demonstrated numerous semineferous tubules that were variable in size and contour. Spermatogonia cells are few with degeneration and the maturation was up to primary spermatocyte. Sertoli cells are numerous and some tubules had desquation and degeneration of the spermatogonia cells. The interstial tissue was focally edematous with congested vessels (Fig. 6 c-1, c-2 and c-3). Histological results of D + L80 group showed numerous semineferous tubules with thin basement membrane. The abnormal configurations are few and the tubules were slightly variable in size and contour. They were lined by spermatogonia cells up to primary spermatogonia. Sertoli cells, spermatids and sperms were present and the interstitial tissue shows no edema or congestion (Fig. 6 d-1, d-2 and d-3). Testis from animals fed with the high dose of lutein (160 mg/kg) showed numerous semineferous tubules, with few scattered abnormal convolutions. The spermatogonia in most of the tubules are degenerated. Some tubules show focal proliferations of the spermatogonia. The tubules are slightly variable in size and contour and the interstitial tissue shows no edema or congestion (Fig. 6 e-1, e-2 and e-3).

### Statistical analysis

Data were expressed as Mean ± SEM. and analyzed using one-way ANOVA followed by Student Newman-Keuls multiple comparisons test (*n* = 6). Difference between groups was considered statistically significant when (*) P ≤ 0.05, (**) P ≤ 0.01 and (***) P ≤ 0.001. (a) D group was compared with ND group; (b) lutein treated groups were compared with D group.

## Discussion

The present investigation outlines the protective effects of lutein against experimentally induced diabetic sexual oxidative dysfunction in Wistar rats. The preventative properties of lutein were confirmed by the histological evaluation. Administration of lutein for five consecutive weeks markedly reduced signs of testicular inflammation, lipid peroxidation, oxidative stress and cellular damage induced by hyperglycemia.

STZ-induced diabetes in rodents appears to be the most suitable animal model because it reflects the symptoms of diabetes in human [24] and it is characterized by severe loss in body weight, and this is also reflected in the present study. The decreases in body and testis weight in diabetic rats showed the loss or degradation of structural proteins due to diabetes and a significant reduction in the serum levels of the main androgenic hormone, testosterone [25]. Indeed, the role of oxidative impairments in the male reproductive system has been suggested in several experimental studies [26–28]. Induction of lipid peroxidation process and elevation of its biomarker like TBARS in the testicular tissues was shown to have fundamental implications on testicular physiology and sperm function [29]. DM is well known to be strongly correlated with oxidative stress leading to production of free radicals and act as intercellular second messengers that can induce activation downstream signaling of many molecules, including transcription factors like nuclear factor kappa B (NF-κB). These NF-κB and other transcription factors mediate vascular smooth muscle cell growth and migration as well as the expression of pro-inflammatory cytokines such as TNF-α, IL-1β, and IL-6 [30]. The present results revealed markedly elevation in the levels of TNF- α and IL-1 β in the diabetic group of animals. These elevated pro-inflammatory mediators antagonize insulin action because of their ability to augment insulin receptor substrate phosphorylation, leading to insulin resistance [31, 32]. Therefore, attenuation of free radical induced NF-κB translocation and ameliorating oxidative stress in diabetic rats explains an associative relationship between the inflammatory cytokines and DM. In the present study, both endogenous antioxidants total and non protein sulfhydryl molecules were found to be inhibited in the diabetic group. Moreover, activities of the antioxidant enzymes SOD and CAT were markedly reduced in the testis of the diabetic animals. The present data demonstrated the significant elevation of lipid peroxidation biomarker MDA in testicular tissues of diabetic animals. These findings are consistent with the increased cellular oxidative stress and accumulation of lipid peroxides levels demonstrated in experimental type-I DM [33]. It is well documented that the endogenous sulfhydryl molecules have intracellular antioxidant properties and several biological functions such as cellular protection against oxidation via a strong nucleophilic action that protects nucleic acids, proteins, and other bio-molecules from ROS [34]. In this regard, a decreased level of T-GSH and NP-SH implicates the reduction of the nucleic acids and TP concentrations in testicular tissues of the diabetic animals reported in our study, which indicates cytotoxicity and oxidative injury of male reproductive organs.

Abundantly antioxidants are present in natural resources and they could have an important influence on human health. Lutein is one of the most widely distributed carotenoids [35] and it is considered as the second most prevalent carotenoid in human serum [36]. Lutein is the only carotenoid that is absorbed in the blood stream after ingestion [35] and accumulated in the human tissue. It showed several biological beneficial properties against different pathological conditions including cell transformation, monocyte-mediated inflammatory response, oxidative stress, LDL oxidation and macula injury [37–40] as well as amelioration of the effects of degenerative human diseases, such as age-related macular degeneration [41, 42] or cataract [43]. Several studies suggested that lutein may help maintain heart health by reducing the risk of atherosclerosis [40, 44]. Lutein treated groups revealed inhibition in diabetes-induced reduction of serum levels of testosterone. In addition that, the histopathological results showed lutein can protect the diabetic associated testicular tubules alterations of their size and contour. Lutein also protected spermatogonia cells from diabetes-induced degeneration, immaturation and congestion.

The structures of lutein is characterized by the presence of two hydroxyl groups, one on each side of the molecule, which are believed to play a critical role in its biologic function [35, 45]. Thus, lutein can be considered as effective quenchers of singlet oxygen and related reactive oxygen species [17, 46]. In addition to quenching ROS directly, lutein was reported to effectively prevent protein, lipid or DNA from oxidative damage by regulating other cellular antioxidant systems [47]. However, in present study, diabetes-induced inhibition in testicular levels of sulfhydryl groups were found to be increased by lutein content dietary supplementation in rats. Similarly, inhibited antioxidant enzymes SOD and CAT activities were also improved following lutein dietary supplementations. Furthermore, the elevated levels of testicular lipid peroxidation product, MDA levels were also markedly lowered by lutein supplementations in the diabetic rats. The cytoprotective effect of lutein was found in the present study as revealed significant improvement in the decreased levels of cellular nucleic acids and total proteins in testicular cells of diabetic rats. It has shown that lutein supplementation effectively blocked the H_2_O_2_-induced protein oxidation, lipid peroxidation and DNA damage in lens of epithelial cells. Moreover, lutein supplementation considered the increase in the levels of GSH and GSSG, particularly after H_2_O_2_ challenge [47]. This finding revealed the ability of lutein for modulating lipid peroxidation and ROS production, it may be suggested through the protective effect of lutein.

Oxidative stress triggers inflammatory process via vicious cycle [48, 49]. However, the regulation of ROS might inhibit the tissue damage caused by inflammatory reactions. In support of this idea, several reports using animal models suggest that the administration of antioxidants reduces ROS and is effective for preventing or treating inflammatory diseases such as rheumatoid arthritis [50], arteriosclerosis [51], vascular changes in diabetes [52] and inflammatory bowel disease [53]. In the present investigation, lutein showed an antiinflammatory effects as it significantly reduced the augmented testicular levels of proinflammatory cytokines like TNF-α and IL-1β. Earlier its antiinflammatory properties have been demonstrated in mammalian systems [54]. Lutein modulated the inflammatory response is suggested to be via protecting the proteasome from inactivation by oxidative stress [55].

## Conclusion

From the above discussion, it may be concluded that lutein has a significant protective effect on testicular dysfunctions noted in STZ induced diabetic state. The actual mechanism for such protection is not clear from this work. However, it may contribute to its antioxidative and antiinflammatory properties. Further investigations are still needed to confirm and evaluate these effects in both experimental and clinical levels.
